# Early Predictors of Surgical Explantation of Transcatheter Aortic Valve Replacement: A Multi-Center International Database Analysis

**DOI:** 10.3390/jcm15041527

**Published:** 2026-02-14

**Authors:** George Bcharah, Sant Kumar, Juan M. Farina, Hend Bcharah, Mahmoud Abdelnabi, Jonathan Sayegh, Ramzi Ibrahim, Omneya Kandil, Hussein Abdul Nabi, Ahmad Jabri, Hursh Naik, Pyong D. Yoon, Bryan Barrus, Kristen A. Sell-Dottin

**Affiliations:** 1 Department of Cardiothoracic Surgery, Mayo Clinic, Phoenix, AZ 85054, USAsell-dottin.kristen@mayo.edu (K.A.S.-D.); 2 Department of Cardiovascular Disease, School of Medicine, Creighton University, Phoenix, AZ 85054, USA; 3 College of Medicine, University of Arizona, Tucson, AZ 85721, USA

**Keywords:** transcatheter aortic valve replacement, explantation, surgical aortic valve replacement, structural heart disease

## Abstract

**Background**: Indications for TAVR explant have been established, although limited data exist regarding pre-TAVR baseline characteristics that predict eventual explantation. **Methods**: The TriNetX network, a database comprising medical records from over 105 institutions, was used. Two cohorts were created: those who underwent TAVR without explant and those requiring subsequent TAVR explant and SAVR. Predictors of explantation were analyzed by multivariate models. **Results**: Among the 63,377 patients undergoing TAVR, 273 (0.4%) required explantation. Patients in the explant group were younger (69.1 ± 11.3 vs. 78.1 ± 8.8 years; *p* < 0.001), more likely to have a thoracic aortic aneurysm (TAA) (10.6% vs. 4.7%; *p* < 0.001) and had higher LDL levels (88.2 ± 41.3 vs. 80.7 ± 34.6 mg/dL; *p* = 0.011). They also had increased post-TAVR rates of acute kidney injury (9.2% vs. 5.2%; *p* = 0.004) and paravalvular leak (5.9% vs. 0.9%; *p* < 0.001). Age at TAVR (HR 1.04; CI 1.03–1.06), baseline TAA (HR 1.69; CI 1.09–2.63), history of infective endocarditis (HR 1.92; CI 1.10–3.35), and higher LDL (HR 1.02; 95% CI 1.00–1.03) were independent predictors for explantation. **Conclusions**: Younger age at TAVR, TAA, history of endocarditis, and elevated baseline LDL were notable predictors of explantation. These findings highlight the necessity of pre-procedural assessment and follow-up in high-risk patients to optimize TAVR durability.

## 1. Introduction

The incidence of transcatheter aortic valve replacement (TAVR) has risen significantly over the past decade, with annual procedure volumes in the United States surpassing surgical aortic valve replacement (SAVR) [1]. Vascular complications can occur in TAVR, particularly in those with peripheral vascular disease, which are associated with increased mortality [2,3]. Paravalvular aortic regurgitation and embolic events due to catheter manipulation of the aorta and valve are also notable risks [3,4]. Although uncommon, surgical valve explantation may be required in some cases [5]. Patients who require valve explantation often present with complications where a TAVR-in-TAVR is not feasible, such as infective endocarditis (IE) or severe paravalvular leak (PVL), or they present for a reintervention where TAVR-in-TAVR is not feasible due to anatomical reasons. In addition to paravalvular regurgitation and embolic events, other serious TAVR-related complications include acute coronary ostial obstruction, aortic root or annular rupture related to heavy valvular or annular calcification, and device malposition or migration, all of which may necessitate urgent surgical intervention or valve explantation [6]. Younger age at the time of TAVR, lower surgical risk, and complex cardiac histories have been associated with increased explantation risk [6,7,8].

While many studies have explored post-TAVR complications that increase the risk of TAVR explantation, no study to date has examined the impact of pre-TAVR baseline characteristics and comorbidities on TAVR explantation. Additionally, the potential influence of short-term post-TAVR outcomes on the need for explantation remains unexplored [5,6,7,8]. This study aims to identify the predictors of TAVR explant by analyzing baseline comorbidities and short-term post-TAVR outcomes.

## 2. Materials and Methods

### 2.1. Study Design and Population

**Figure 1:**
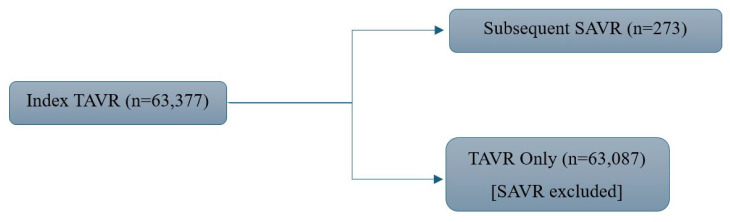
Study cohort. Flowchart illustrating patient selection within the TriNetX database among 63,377 patients who underwent transcatheter aortic valve replacement (TAVR).

This was a retrospective cohort study conducted using TriNetX, a global federated health research network that provides de-identified electronic medical records (including diagnoses, procedures, medications, and laboratory values) from 105 participating healthcare organizations (HCOs) grouped into a network called “Research.” Data contributions are harmonized using a standard, research-ready terminology mapping framework that includes ICD-9/ICD-10 for diagnoses and inpatient procedures, CPT (and in some cases ICD-9/10-PCS) for outpatient and procedural billing, LOINC (Logical Observation Identifiers, Names and Codes) for clinical tests, and RxNorm (RxNorm Concept Unique Identifier) for medications. Notably, while CPT codes are supported within the data, their usage—and completeness—may vary by region. In particular, coding practices outside the U.S. may rely more heavily on alternate or local procedure coding systems, meaning that CPT-coded procedure identification may underrepresent non-U.S. sites. This database has demonstrated reliable utility to address real-world research questions, generating over 1500 publications since 2018 [9]. Within TriNetX, two cohorts of adult patients (≥18 years of age) were defined and compared: one in which patients underwent a TAVR but did not receive a subsequent surgical valve explant and another in which patients underwent TAVR followed by surgical aortic valve replacement (Figure 1). These patients were identified using Current Procedural Terminology (CPT) codes. Data were extracted until July 2025. All data obtained were de-identified and, as such, did not require institutional review board approval.

### 2.2. Data Collection

Demographics (age, sex, race/ethnicity), comorbidities (e.g., diabetes mellitus, chronic kidney disease, atrial fibrillation, thoracic aortic aneurysm, etc.), and select laboratory values (cardiac biomarkers, cholesterol panel, inflammatory markers, hemoglobin A1c) were extracted and compared between groups. A history of prior infective endocarditis was defined as the presence of an ICD-9 or ICD-10 diagnosis code for infective endocarditis recorded at any time before the index TAVR procedure. Outcomes collected included presence of complete heart block, acute kidney injury, stroke, prosthesis complications, bleeding, prolonged ventilation, pacemaker insertion, respiratory failure, cardiogenic shock, pneumonia, sepsis, aortic root repair, and death. All outcomes above are defined per their coding descriptions in Supplemental Table S1. Prosthesis complications were identified using ICD-10 coding definitions within the TriNetX platform, which categorizes events such as valve breakdown, displacement, or leakage; while this coding approach may not capture the full spectrum of clinically recognized prosthetic valve complications, it reflects the terminology available for data extraction in this database. All ICD-10 and CPT codes used for outcomes are outlined in Supplemental Table S1.

### 2.3. Index Event and Outcomes Timing

For each cohort, the index event was defined as the date of the first TAVR procedure when comparing baseline characteristics and 30-day post-TAVR outcomes. For patients who did not require explantation, outcomes were evaluated within 30 days following the index TAVR procedure. For patients who ultimately required surgical explantation, outcomes were analyzed in two distinct ways: (1) short-term events occurring within 30 days of the index TAVR, to allow comparison with the non-explant cohort, and (2) complications occurring after the explant procedure, assessed at 30 days, 3 months, 6 months, 1 year, and 3 years following the explant. This distinction ensures that “post-TAVR” outcomes reflect early complications after the index procedure, while “post-explant” outcomes reflect events specifically after surgical reintervention.

### 2.4. Statistical Analysis

All analyses were performed using TriNetX’s built-in “Compare Outcomes” analytic suite. Baseline characteristics were summarized with descriptive statistics (means ± standard deviation for continuous variables and percentages for categorical variables), and differences between groups were assessed using appropriate statistical tests (e.g., *t* test, chi-square test).

Hazard ratios (HRs) and 95% confidence intervals (CIs) were estimated using a Cox proportional hazards model available in TriNetX to quantify associations between candidate predictors and the subsequent risk of valve explantation. All baseline variables were first assessed in univariable Cox proportional hazards regression. Variables with *p* < 0.10, together with clinically relevant variables such as age and sex, were included in the multivariable Cox regression model, as this is the only multivariable method available within the TriNetX platform. A *p*-value < 0.05 was deemed statistically significant. All statistical analyses were conducted in TriNetX, as data could not be extracted for external software analysis.

## 3. Results

### 3.1. Baseline Characteristics

A total of 63,377 patients underwent TAVR. The average age of the overall population was 78.1 ± 8.83 years, where 40% were females (Table 1). Among the 63,377 patients, 273 (0.4%) required valve explantation. Compared to patients who did not require explantation, those who did were younger (69.1 ± 11.3 years vs. 78.1 ± 8.82 years; *p* < 0.001), more likely to be Hispanic or Latino (7.3% vs. 3.5%; *p* = 0.001), and had a higher baseline body surface area (2.1 ± 0.7 vs. 2.00 ± 0.3, *p* = 0.038). Patients undergoing valve explantation were also less likely to have chronic kidney disease (22.3% vs. 31.5%; *p* = 0.001) or coronary artery disease (CAD) (68.1% vs. 73.9%; *p* = 0.030) and had lower rates of prior percutaneous coronary intervention (10.9% vs. 15.1%, *p* < 0.001) but were more likely to have atrial fibrillation (55.7% vs. 36.4%; *p* < 0.001) and thoracic aortic aneurysms (10.6% vs. 4.7%; *p* < 0.001). Patients who required explantation had significantly higher levels of total cholesterol (163 ± 56.0 mg/dL vs. 152 ± 44.8 mg/dL; *p* = 0.006) and LDL cholesterol (88.2 ± 41.3 mg/dL vs. 80.7 ± 34.6 mg/dL; *p* = 0.011).

### 3.2. Post-TAVR Outcomes

Outcomes in Table 2 reflect events occurring within 30 days of the index TAVR procedure. These analyses compare patients who eventually required explantation with those who did not. Patients who required TAVR explantation down the line were significantly more likely to experience acute kidney injury (9.2% vs. 5.2%; *p* = 0.004), bleeding (17.2% vs. 6.6%; *p* < 0.001), and paravalvular leak (5.9% vs. 0.9%; *p* < 0.001) within 30 days of undergoing the index TAVR (Table 2).

### 3.3. Post-Explantation Outcomes

Outcomes in Table 3 reflect complications occurring after surgical explantation, with follow-up intervals measured from the date of the explant procedure (30 days, 3 months, 6 months, 1 year, and 3 years). At 30 days, the most prevalent complications in the explant group, outside of arrhythmias (46.5%), were bleeding (26.7%), acute kidney injury (20.5%), and respiratory failure (19.8%), with mortality at 8.4% and prosthesis-related complications at 4.8% (Table 3). Mortality continued to increase, nearly doubling to 13.6% in 6 months and rising to 18.7% by 3 years. Prosthesis complications continued to slowly rise across the outcome time intervals, reaching 7.7% by 3 years.

### 3.4. Predictors of TAVR Explantation

**Figure 2:**
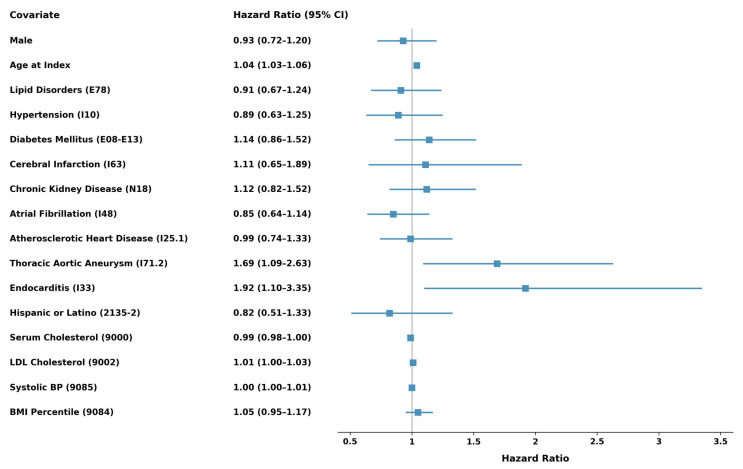
Predictors of TAVR explant. Forest plot demonstrating independent predictors of surgical explantation after TAVR derived from multivariable Cox proportional hazards analysis.

Multivariate analysis identified age at the time of TAVR (HR: 1.04; CI: 1.03–1.06; *p* < 0.001) and a history of thoracic aortic aneurysm (TAA) (HR: 1.69; CI: 1.09–2.63; *p* = 0.020) as independent predictors of valve explant (Figure 2). Other independent predictors of valve explant included a history of pre-TAVR endocarditis (HR: 1.92; 95% CI: 1.10–3.35; *p* = 0.022) and serum LDL levels (HR: 1.02; 95% CI: 1.00–1.03; *p* = 0.007).

## 4. Discussion

Transcatheter aortic valve replacement explantation has been reported as the fastest growing cardiac surgery procedure in the United States [10]. Multiple studies over the past two decades have explored trends in TAVR explantation, and it has been characterized as a rare complication of TAVR, especially in earlier reports. A Society of Thoracic Surgeons (STS) database study identified only 123 explant cases across U.S. centers from 2011 to 2015, during the times when TAVR was limited to high-risk patients [11]. An analysis of Medicare patients from 2012 to 2017 (132,633 TAVR cases) also found an explant incidence of about 0.2% [5]. The rate was higher in the early TAVR era (~0.28% up to 2015) and lower in the more recent era (~0.14%), possibly suggesting that improvements in technology and patient selection may have reduced early failures. In a single-center analysis, a similar low incidence (0.2%) was reported in the 2013–2016 period, rising to 0.7% in 2017–2020 as TAVR usage expanded [12]. A statewide TAVR registry in Michigan observed an overall explant frequency of 0.4% [13]. Recent international registries (EXPLANT-TAVR) collected 401 cases of TAVR explants (391 analyzed) from 42 centers in the U.S. and Europe [7]. All sources emphasize that while the incidence remains low, it is expected to rise as TAVR is increasingly performed in younger, lower-risk patients with longer life expectancy, a concerning trend underscored by recent data showing some centers performing primary TAVR in more than 40% of patients under the age of 65 [1,5]. Consistent with this, patients undergoing TAVR explant in the present cohort tend to be younger (mean age 69.1 vs. 78.1 years) and relatively lower-risk compared to the non-explant group. In the Medicare cohort, explant patients had fewer comorbidities, such as heart failure, and a greater proportion were initially in a low-risk surgical category [5]. This is likely a reflection of the patient selection process—healthier, younger patients not only have longer to live (i.e., more time for a TAVR to degenerate), but are also more likely to be offered surgery if the TAVR fails, whereas older, high-risk patients might not be candidates for explant. Consequently, multiple series noted that patients’ surgical risk scores were higher at the time of explant than at the original TAVR, which could reflect clinical deterioration or more complex pathology by the time surgery was needed [12,14]. While previous studies outline complications in the post-TAVR and pre-explant phase, the present study is the first to examine pre-TAVR characteristics and their impact on the need for eventual TAVR explant.

Importantly, while patients who ultimately underwent explantation were younger at baseline, increasing age emerged as an independent predictor of explantation in the multivariable Cox model. This apparent discrepancy likely reflects differences between crude cross-sectional comparisons and adjusted time-to-event analyses. Older patients have substantially higher competing mortality risks following TAVR and may die before structural valve deterioration or other complications necessitate reintervention. In addition, advanced age and frailty may limit candidacy for surgical explant, leading to the preferential use of valve-in-valve TAVR or conservative management. Thus, the Cox model reflects the adjusted hazard among patients who remain alive and eligible for surgery, whereas the baseline comparison reflects the overall demographic profile of patients who ultimately underwent explant.

These findings are particularly relevant in the context of contemporary guideline-directed decision-making for aortic valve replacement, which increasingly emphasizes patient age, life expectancy, and anticipated need for future reintervention. Contemporary guidelines emphasize an age- and life expectancy-based “lifetime management” approach to selecting SAVR versus TAVR. The 2020 American College of Cardiology/American Heart Association (ACC/AHA) [15] and 2021 European Society of Cardiology (ESC/EACTS) [16] guidelines generally favor SAVR in younger, lower-risk patients with longer life expectancy, recommend either SAVR or transfemoral TAVR in intermediate-age groups following shared decision-making, and favor transfemoral TAVR in older patients or those with limited life expectancy when anatomy is suitable. These recommendations reflect not only the peri-procedural risk but also the anticipated need for future reintervention, as younger patients are more likely to outlive an initial bioprosthesis. Accordingly, the feasibility and long-term implications of redo strategies—such as valve-in-valve TAVR versus surgical explant and replacement—should be carefully weighed, particularly given concerns related to cumulative gradients, coronary access, and the potential need for complex surgery after multiple transcatheter valves.

A history of pre-TAVR infective endocarditis emerged as the most significant risk factor for subsequent surgical explant in the cohort. Current guidelines from the ACC/AHA [15] and the ESC [16] discourage TAVR intervention in the setting of active IE, although a few reports have described its use in IE where surgical intervention was prohibitively high-risk [17,18]. Santos-Martínez et al. in a multicenter retrospective study reported on TAVR use in patients with residual aortic valve dysfunction after successfully treated IE [19], and Brankovic et al. in a systematic review evaluated six case reports where TAVRs were successfully done as “rescue therapy” in certain high-risk cases [20]. However, long-term data in these rare, highly select scenarios have not been explored, and the present study suggests that explantation may be one of the long-term complications to consider with TAVR use in IE.

The available data suggest that the overall incidence of infective endocarditis after TAVR and SAVR is similar, generally ranging from approximately 0.3 to 1.2% per patient-year for both approaches [15,16]. While large observational studies and meta-analyses have not demonstrated a consistent difference in overall endocarditis rates between TAVR and SAVR, important distinctions exist in clinical presentation and outcomes. TAVR-associated endocarditis more frequently involves enterococcal organisms, prosthetic valve infection, and peri-annular complications, and is associated with higher mortality, in part due to advanced patient age, comorbidity burden, and limited surgical candidacy. Current ACC/AHA and ESC/EACTS guidelines do not recommend one valve replacement strategy over the other solely to reduce endocarditis risk; rather, procedural selection should be guided by patient age, surgical risk, anatomy, and life expectancy [15,16]. In patients with a history of prior infective endocarditis, careful Heart Team evaluation is warranted, as long-term durability and the potential need for future surgical intervention may favor SAVR in selected younger, lower-risk patients.

Thoracic aortic aneurysm was also a strong predictor of TAVR explant, and several mechanisms could explain why it predisposes to TAVR failure. An aneurysmal ascending aorta may alter the valve geometry and can lead to malalignment, increased shear stress, or late annular enlargement around the TAVR prosthesis [21,22]. The presence of a TAA can also indicate an intrinsic tissue weakness and can be associated with underlying connective tissue disorders (e.g., bicuspid aortic valve with aortopathy), potentially impacting bioprosthetic valve durability [22,23]. The rates of bicuspid aortic valve were higher in the explant group in the present cohort (7.9% vs. 2.0%, *p* = 0.282) although they lacked significance, and further studies are needed to investigate any associations. However, it is also plausible that the association with higher explantation rates reflects a lower clinical threshold to proceed with surgery in these patients, as the presence of a concomitant aneurysm may influence clinicians to opt for earlier intervention, including aneurysm repair. Additionally, progressive growth of the aneurysm itself may directly necessitate intervention, further contributing to increased explantation rates. From a management standpoint, structural or functional TAVR failure in patients with significant aneurysms typically necessitates surgical repair rather than a valve-in-valve redo due to anatomical constraints or procedural risks [24]. Where feasible, surgical valve replacement with an aortic root repair, when indicated, during the index procedure may be the preferable strategy for such patients, especially if they are reasonable surgical candidates. For high-risk patients who undergo TAVR despite a TAA, close surveillance of the aneurysm is critical, and earlier referral for elective surgical correction may be indicated if the aneurysm progresses.

Structural valve deterioration was the single most common indication for TAVR explant in many studies. For example, 79% of cases in one series were due to bioprosthetic failure [5]. In the international EXPLANT-TAVR registry, roughly 25–30% of failures were SVD, similar across valve types [7]. In the STS early cohort, 15% had paravalvular leak and another ~22% had technical issues (malposition, sizing, device migration, etc.) as the reason for reoperation [11]. Michigan data likewise showed procedure-related failures (including paravalvular regurgitation) accounting for about 35% of explants, typically occurring shortly after TAVR (median ~4–7 months) [13]. Risk factors such as metabolic syndrome, diabetes, and dyslipidemia, including higher levels of lipoprotein(a) and LDL/HDL ratios, have been strongly associated with accelerated SVD in bioprostheses [25]. This could explain why the explant cohort had higher LDL levels on baseline. Although higher baseline LDL was independently associated with explantation, the magnitude of the hazard ratio was modest. This finding likely reflects LDL as a cumulative, long-term contributor to bioprosthetic valve degeneration rather than a short-term determinant of failure. Accordingly, while statistically significant, the effect size should be interpreted with caution and viewed as hypothesis-generating rather than determinative at the individual patient level. Biologically, LDL cholesterol can accelerate the calcific degeneration of the bioprosthetic valve leaflets by infiltrating the extracellular matrix, promoting oxidative stress and inflammatory cascades similar to those seen in native valve disease [25]. This promotes calcium deposition and fibrous thickening in the leaflets, which over time leads to valvular stenosis and/or regurgitation. It is also possible that higher LDL is a surrogate for other unmeasured factors (e.g., lower use of statins or other cardioprotective medications, or a propensity for more aggressive disease) that in turn drive earlier failure. Notably, prior studies of surgical bioprosthetic valves suggested that statin therapy might slow leaflet calcification, although the results have been mixed and lack long-term follow-up [26]. Our findings reinforce the relationship between cholesterol levels and valve outcomes, suggesting more aggressive lipid-lowering therapy after TAVR to improve prosthetic valve durability, although further investigations are indicated.

Survivors of TAVR explant often endure high rates of complications. In Michigan’s report, 76% of patients had at least one in-hospital complication [13]. According to the meta-analysis, the pooled incidence of stroke was about 5%, and acute kidney injury (often requiring dialysis) about 16% [27]. Conduction disturbances requiring permanent pacemaker implantation occurred in approximately 13% of patients, likely due to the extensive annular decalcification or root work during explant. Additionally, about 12% of patients were readmitted within 30 days post-discharge. In short, morbidity is very high, and even those who survive to discharge often require rehabilitation—only 37% were able to go directly home in the Michigan series [13]. Nevertheless, rates of complications are steadily improving as operator experience grows and procedural techniques continue to evolve. For example, data from the STS/ACC TVT registry showed a downward trend in permanent pacemaker implantation rates from 2016 to 2020 [28].

TAVR has traditionally been performed in patients at increased or prohibitive surgical risk, which strongly influences reintervention strategies following TAVR-related complications. In this population, redo-TAVR (valve-in-valve) is often preferred when anatomically feasible, whereas surgical explantation is typically reserved for complications such as infective endocarditis, severe paravalvular leak, valve malposition, coronary obstruction, or aortic injury that are not amenable to transcatheter treatment despite higher operative risk. These considerations highlight the importance of lifetime management planning and careful patient selection at the time of the index procedure.

Particularly challenging is when the TAVR device is embedded in the aortic root: surgeons sometimes must perform an aortic root replacement or patch repair of the aorta due to injury from the explant. In one contemporary series, about 20–24% required a full root replacement during the explant, and 9–22% required replacement of the ascending aorta (with the higher frequency in cases of self-expanding valve removal) [29]. The need for these extensive reconstructions further adds to operative risk. An international registry found that if a concomitant mitral valve surgery was needed, it independently doubled the hazard of 1-year mortality (HR ~2.0) [7]. Despite these risks, conditional 1 year survival remains reasonably good, averaging around 75–80% across several large studies. For example, the Medicare cohort had a 1-year mortality of 22.9% [5], (i.e., ~77% survival to one year). The international EXPLANT-TAVR registry likewise found no difference in 1-year survival between valve types, implying a similar overall one-year outcome on the order of 77.1%. Smaller series have reported somewhat lower one-year survival (the Leipzig group saw ~66.5% at 1 year, likely due to a high proportion of endocarditis cases) [12]. Thus, our findings represent a step toward identifying patients at a higher risk for surgical explantation after TAVR. Future investigations directly comparing redo-TAVR versus surgical explant cohorts will be critical to determine how the predictors of reintervention influence procedural selection, durability, and long-term outcomes.

## 5. Study Limitations

The findings of this study need to be taken in the context of its limitations. This was a retrospective cohort study which introduces inherent biases in patient selection and data collection. Unmeasured confounders that were not available in the database (e.g., genetic factors, variations in operative technique, or differences in post-TAVR medical therapy) could influence the likelihood of explantation. Some potentially important predictors might not have reached significance due to the limited sample size. In addition, data on the type of TAVR valve used (e.g., balloon-expandable vs. self-expanding) were not available, preventing a more extensive analysis of whether the valve type influenced the risk of explantation. Information regarding the specific indication for valve explantation and the time interval between the index TAVR and the explant procedure was also unavailable, which limits the ability to investigate whether certain baseline comorbidities were associated with particular indications for explantation, such as SVD or IE. Moreover, the timing, severity, microbiology, and confirmation of resolution of prior infective endocarditis could not be determined, and therefore this variable should be interpreted as a historical diagnosis rather than a granular clinical phenotype. The TriNetX database does not allow for a regional-based comparison, which limits the analysis of explant rates and outcomes across institutions and regions. Furthermore, formal adjustments for multiple comparisons were not applied to the univariate analysis of baseline characteristics due to the constraints of the analytics platform; therefore, these comparisons should be considered exploratory. Lastly, baseline Society of Thoracic Surgeons (STS) risk scores for perioperative morbidity and mortality were not available in the TriNetX database, which prevented assessment of whether surgical risk stratification influenced the likelihood of explantation. Furthermore, the definition of “prosthesis complications” was based on ICD-10 coding within the TriNetX database (valve breakdown, displacement, or leakage), which may not fully correspond to the way prosthetic valve complications are typically described in clinical practice. As such, these findings should be interpreted with caution, recognizing the inherent limitations of coding-based outcome classification.

## 6. Conclusions

In conclusion, this study identifies critical predictors and post-procedural complications associated with TAVR explantation, including infective endocarditis, thoracic aortic aneurysm, and elevated LDL levels before index TAVR. While increasing age was associated with explantation in time-to-event analysis, this finding likely reflects competing mortality risk and differences in surgical candidacy rather than a simple demographic effect. Similarly, although elevated LDL was statistically associated with explant, the magnitude of association was modest and should be interpreted as a potential long-term contributor to bioprosthetic degeneration rather than a short-term determinant. These findings underscore the importance of detailed pre-procedural evaluation and close post-procedural monitoring to mitigate risks and improve patient outcomes. Further research is needed to better understand the underlying mechanisms driving these associations and to refine patient selection criteria and management strategies for TAVR recipients.

## Figures and Tables

**Table 1:** Baseline characteristics across the explant and non-explant cohorts.

	Explant Cohort (*n* = 273)	Non-Explant Cohort (*n* = 63,104)	*p*-Value
Age, years (mean ± standard deviation)	69.1 ± 11.3	78.1 ± 8.82	<0.001
Female, *n* (%)	98 (35.9)	25,500 (40.4)	0.130
Ethnicity and race			
Hispanic or Latino, *n* (%)	20 (7.3)	2230 (3.5)	0.001
White, *n* (%)	208 (76.2)	50,413 (79.9)	0.128
Black/African American, *n* (%)	15 (5.5)	2161 (3.4)	0.061
Hypertension, *n* (%)	201 (73.6)	48,072 (76.2)	0.323
Hyperlipidemia, *n* (%)	166 (60.8)	40,766 (64.6)	0.191
BMI ≥ 30, *n* (%)	34 (12.5)	8719 (13.8)	0.515
Diabetes mellitus, *n* (%)	98 (35.9)	23,320 (37.0)	0.718
Prior stroke, *n* (%)	17 (6.2)	5139 (8.1)	0.248
Chronic obstructive pulmonary disease, *n* (%)	46 (16.8)	12,570 (19.9)	0.205
Pulmonary hypertension, *n* (%)	27 (3.0)	869 (1.4)	0.108
Pectus excavatum, *n* (%)	0 (0.0%)	45 (0.1)	0.036
Asthma, *n* (%)	31 (11.4)	6106 (9.7)	0.349
Chronic kidney disease, *n* (%)	61 (22.3)	19,875 (31.5)	0.001
Atrial fibrillation, *n* (%)	152 (55.7)	22,963 (36.4)	<0.001
Ventricular tachycardia, *n* (%)	23 (8.4)	3730 (5.9)	0.079
Coronary artery disease, *n* (%)	186 (68.1)	46,640 (73.9)	0.030
Peripheral vascular disease, *n* (%)	39 (14.3)	10,533 (16.7)	0.287
Prior endocarditis, *n* (%)	17 (6.2)	660 (1.0)	<0.001
Prior percutaneous coronary intervention, *n* (%)	30 (10.9)	9529 (15.1)	<0.001
Malignancy, *n* (%)	122 (44.7)	22,626 (35.9)	0.002
Thoracic aortic aneurysm, *n* (%)	29 (10.6)	2986 (4.7)	<0.001
Atherosclerosis of aorta, *n* (%)	79 (29.1)	13,911 (22.0)	0.267
Bicuspid aortic valve, *n* (%)	21 (7.9)	1286 (2.0)	0.282
Laboratory data			
Albumin, g/dL	3.89 ± 0.506	3.98 ± 0.482	0.003
Troponin I, ng/mL (mean ± standard deviation)	1.29 ± 7.92	0.884 ± 7.81	0.703
BNP, pg/mL (mean ± standard deviation)	772 ± 1810	1125 ± 3971	0.330
NT-proBNP, pg/mL (mean ± standard deviation)	3146 ± 5544	4519 ± 8915	0.258
Hemoglobin A1c, % (mean ± standard deviation)	6.2 ± 1.4	6.15 ± 1.31	0.624
Total cholesterol, mg/dL (mean ± standard deviation)	163 ± 56.0	152 ± 44.8	0.006
LDL, mg/dL (mean ± standard deviation)	88.2 ± 41.3	80.7 ± 34.6	0.011
HDL, mg/dL (mean ± standard deviation)	46.8 ± 19.4	47.3 ± 19	0.761
ESR, mg/dL (mean ± standard deviation)	31.9 ± 31.5	29.5 ± 25.9	0.490
CRP, mg/dL (mean ± standard deviation)	40.9 ± 69.4	28.7 ± 51.9	0.089
Body surface area, m^2^	2.1 ± 0.7	2.00 ± 0.3	0.038

Abbreviations: BNP = brain natriuretic peptide; NT-proBNP = N-terminal pro b-type natriuretic peptide; LDL = low-density lipoprotein; HDL = high-density lipoprotein; ESR = erythrocyte sedimentation rate; CRP = C-reactive protein.

**Table 2:** 30-day outcomes following index TAVR in patients who required an explant compared to those who did not.

	Explant Cohort (*n* = 273)	Non-Explant Cohort (*n* = 63,104)	*p*-Value
Complete Heart Block	22 (8.1%)	4579 (7.3%)	0.61
Acute Kidney Injury	25 (9.2%)	3298 (5.2%)	0.004
Stroke	12 (4.4%)	1934 (3.1%)	0.203
Prosthesis Complications	36 (13.2%)	213 (0.3%)	<0.001
Bleeding	47 (17.2%)	4191 (6.6%)	<0.001
Ventilator Dependent	11 (4.0%)	442 (0.7%)	<0.001
Pacemaker Insertion	21 (7.7%)	4537 (7.2%)	0.748
Respiratory Failure	23 (8.4%)	2127 (3.4%)	<0.001
Cardiogenic Shock	14 (5.1%)	645 (1.0%)	<0.001
Pneumonia	13 (4.8%)	1617 (2.6%)	0.022
Sepsis	12 (4.4%)	657 (1.0%)	<0.001
Root Repair	<10 (3.7%) *	13 (0.0%)	<0.001
Arrhythmia	97 (35.5%)	19,952 (31.6%)	0.165
Other Shock	<10 (3.7%) *	451 (0.7%)	<0.001
Death	<10 (3.7%) *	998 (1.6%)	0.006

* TriNetX does not report counts of <10.

**Table 3:** Post-TAVR explantation outcomes at 30 days, 3 months, 6 months, 1 year, and 3 years.

Outcome	*n* (%)
**30 Days**	
Complete Heart Block	36 (13.2%)
Acute Kidney Injury	56 (20.5%)
Stroke	22 (8.1%)
Prosthesis Complications	13 (4.8%)
Bleeding	73 (26.7%)
Ventilator Dependent	19 (7.0%)
Pacemaker Insertion	33 (12.1%)
Respiratory Failure	54 (19.8%)
Cardiogenic Shock	31 (11.4%)
Pneumonia	19 (7.0%)
Sepsis	22 (8.1%)
Root Repair	≤10 (≤3.7%) *
Arrhythmia	127 (46.5%)
Other Shock	12 (4.4%)
Death	23 (8.4%)
**3 Months**	
Prosthesis Complications	16 (5.9%)
Death	33 (12.1%)
**6 Months**	
Prosthesis Complications	16 (5.9%)
Death	37 (13.6%)
**1 Year**	
Prosthesis Complications	19 (7.0%)
Death	42 (15.4%)
**3 Years**	
Prosthesis Complications	21 (7.7%)
Death	51 (18.7%)

* TriNetX does not report counts of <10.

## Data Availability

The data presented in this study are available on request from the corresponding author due to privacy and ethical restrictions.

## Supplementary Materials

The following supporting information can be downloaded at https://www.mdpi.com/article/10.3390/jcm15041527/s1, Table S1: ICD/CPT Codes Used for Outcomes of Interest.
