# Supplementary material for: Early Predictors of Surgical Explantation of Transcatheter Aortic Valve Replacement: A Multi-Center International Database Analysis
Source: J Clin Med. 2026 Feb 14;15(4):1527. doi: 10.3390/jcm15041527 (PMC12942133; doi:10.3390/jcm15041527)
Supplement: Supplementary file 1 [file jcm-15-01527-s001.zip › jcm-4131035-supplementary.pdf]

Supplemental Table S1. ICD/CPT Codes Used for Outcomes of Interest

Outcome	Code	Description
Complete Heart Block	ICD10CM:I44.2	Atrioventricular block, complete
Acute Kidney Injury	ICD10CM:N17	Acute kidney failure
Stroke	ICD10CM:I63	Cerebral infarction
Prosthesis Complications	ICD10CM:T82.0	Mechanical complication of heart valve prosthesis
Bleeding	ICD10CM:R58	Hemorrhage, not elsewhere classified
	ICD10CM:T82.83	Hemorrhage due to cardiac and vascular prosthetic devices, implants and grafts
	ICD10CM:D62	Acute posthemorrhagic anemia
	ICD10CM:I31.2	Hemopericardium, not elsewhere classified
	ICD10CM:I23.0	Hemopericardium as current complication following acute myocardial infarction
	ICD10CM:S26.0	Injury of heart with hemopericardium
	ICD10CM:D68.3	Hemorrhagic disorder due to circulating anticoagulants
	ICD10CM:I60	Nontraumatic subarachnoid hemorrhage
	ICD10CM:I61	Nontraumatic intracerebral hemorrhage
	ICD10CM:I62	Other and unspecified nontraumatic intracranial hemorrhage
	ICD10CM:I62.0	Nontraumatic subdural hemorrhage
	ICD10CM:I62.00	Nontraumatic subdural hemorrhage, unspecified
	ICD10CM:I62.9	Nontraumatic intracranial hemorrhage, unspecified
	ICD10CM:K92.2	Gastrointestinal hemorrhage, unspecified
	ICD10CM:R04.2	Hemoptysis
	ICD10CM:R04	Hemorrhage from respiratory passages
	ICD10CM:T82.838	Hemorrhage due to vascular prosthetic devices, implants and grafts
	ICD10CM:T82.838A	Hemorrhage due to vascular prosthetic devices, implants and grafts, initial encounter
	ICD10CM:J94.2	Hemothorax
	ICD10CM:S27.1	Traumatic hemothorax
Ventilator Dependent	ICD10CM:Z99.11	Dependence on respirator [ventilator] status
	ICD10PCS:5A1945Z	Respiratory Ventilation, 24-96 Consecutive Hours
Pacemaker Insertion	ICD10PCS:0JH604Z	Insertion of Pacemaker, Single Chamber into Chest Subcutaneous Tissue and Fascia, Open Approach

	ICD10PCS:0JH634Z	Insertion of Pacemaker, Single Chamber into Chest Subcutaneous Tissue and Fascia, Percutaneous Approach
	CPT:33207	Insertion of new or replacement of permanent pacemaker; ventricular
	CPT:33206	Insertion of new or replacement of permanent pacemaker; atrial
	CPT:33208	Insertion of new or replacement of permanent pacemaker; atrial and ventricular
	CPT:33274	Transcatheter insertion of leadless pacemaker, right ventricular
	CPT:1006079	Insertion of permanent pacemaker with transvenous electrode(s)
Respiratory Failure	ICD10CM:J96.0	Acute respiratory failure
	ICD10CM:J96.2	Acute and chronic respiratory failure
	ICD10CM:J96.9	Respiratory failure, unspecified
Cardiogenic Shock	ICD10CM:R57.0	Cardiogenic shock
	ICD10CM:T81.11	Postprocedural cardiogenic shock
Pneumonia	ICD10CM:J12	Viral pneumonia
	ICD10CM:J13	Pneumonia due to Streptococcus pneumoniae
	ICD10CM:J14	Pneumonia due to Hemophilus influenzae
	ICD10CM:J15	Bacterial pneumonia
	ICD10CM:J16	Pneumonia due to other infectious organisms
	ICD10CM:J18	Pneumonia, unspecified organism
Sepsis	ICD10CM:T81.44	Sepsis following a procedure
	ICD10CM:T81.44XA	Sepsis following a procedure, initial encounter
	ICD10CM:A41	Other sepsis
	ICD10CM:A40	Streptococcal sepsis
Root Repair	CPT:33858	Ascending aorta graft for aortic dissection
	CPT:33859	Ascending aorta graft for aortic disease other than dissection
	CPT:33853	Repair of hypoplastic/interrupted aortic arch
	CPT:33860	Ascending aorta graft (deprecated 2021)
	CPT:1035651	Ascending aorta graft with valve suspension
	CPT:33863	Aortic root replacement with valved conduit
	CPT:33864	Valve-sparing aortic root remodeling
Arrythmia	ICD10CM:I48	Atrial fibrillation and flutter
	ICD10CM:I49	Other cardiac arrhythmias

Other Shock	ICD10CM:R57.1	Hypovolemic shock
	ICD10CM:R57.8	Other shock
	ICD10CM:R57.9	Shock, unspecified
